# Microglial activation mediates chronic mild stress-induced depressive- and anxiety-like behavior in adult rats

**DOI:** 10.1186/s12974-018-1054-3

**Published:** 2018-01-17

**Authors:** Ya-Lin Wang, Qiu-Qin Han, Wen-Qing Gong, Dong-Hui Pan, Li-Zheng Wang, Wei Hu, Min Yang, Bing Li, Jin Yu, Qiong Liu

**Affiliations:** 1 Department of Integrative Medicine and Neurobiology, School of Basic Medical Sciences, Institutes of Brain Science, State Key Laboratory of Medical Neurobiology and Collaborative Innovation Center for Brain Science, Shanghai Medical College, Fudan University, Shanghai, 200032 China; 2 Department of Anatomy, Histology and Embryology, School of Basic Medical Sciences, Shanghai Medical College, Fudan University, Shanghai, 200032 China; 3 Key Laboratory of Medical Imaging Computing and Computer Assisted Intervention of Shanghai, Shanghai, China; 4 Molecular Imaging Center, Jiangsu Institute of Nuclear Medicine, Wuxi, Jiangsu China; 5 Center Laboratories, Jinshan Hospital, Fudan University, Shanghai, 201508 China

**Keywords:** Depression, Microglia, Inflammation, NLRP3 inflammasome, Minocycline

## Abstract

**Background:**

Depression is a heterogeneous disorder, with the exact neuronal mechanisms causing the disease yet to be discovered. Recent work suggests it is accompanied by neuro-inflammation, characterized, in particular, by microglial activation. However, microglial activation and its involvement in neuro-inflammation and stress-related depressive disorders are far from understood.

**Methods:**

We utilized multiple detection methods to detect the neuro-inflammation in the hippocampus of rats after exposure to chronic mild stress (CMS). Male Sprague Dawley (SD) rats were subjected to chronic mild stressors for 12 weeks. Microglial activation and hippocampal neuro-inflammation were detected by using a combinatory approach of in vivo [18F] DPA-714 positron emission computed tomography (PET) imaging, ionized calcium-binding adapter molecule 1 and translocator protein (TSPO) immunohistochemistry, and detection of NOD-like receptor protein 3 (NLRP3) inflammasome and some inflammatory mediators. Then, the rats were treated with minocycline during the last 4 weeks to observe its effect on hippocampal neuro-inflammation and depressive-like behavior induced by chronic mild stress.

**Results:**

The results show that 12 weeks of chronic mild stress induced remarkable depressive- and anxiety-like behavior, simultaneously causing hippocampal microglial activation detected by PET, immunofluorescence staining, and western blotting. Likewise, activation of NLRP3 inflammasome and upregulation of inflammatory mediators, such as interleukin-1β (IL-1β), IL-6, and IL-18, were also observed in the hippocampus after exposure to chronic stress. Interestingly, the anti-inflammatory mediators, such as IL-4 and IL-10, were also increased in the hippocampus following chronic mild stress, which may hint that chronic stress activates different types of microglia, which produce pro-inflammatory cytokines or anti-inflammatory cytokines. Furthermore, chronic minocycline treatment alleviated the depressive-like behavior induced by chronic stress and significantly inhibited microglial activation. Similarly, the activation of NLRP3 inflammasome and the increase of inflammatory mediators were not exhibited or significantly less marked in the minocycline treatment group.

**Conclusion:**

These results together indicate that microglial activation mediates the chronic mild stress-induced depressive- and anxiety-like behavior and hippocampal neuro-inflammation.

## Background

Major depressive disorder (MDD) is a type of neurological disorder that has the following manifestations: loss of interest in activities, feeling lethargic or deprived of energy and grit, disturbed sleep pattern and appetite, low self-esteem, and thoughts of or attempts at suicide [1]. In today’s society, MDD is arguably a grave public health concern, and it has increased the global burden of disease. To present, it has affected an estimated 16% of the entire population [2]. Depression is speculated to be the main attributor responsible for the burden of worldwide non-fatal diseases and also the fourth prime contributor of worldwide disease burden by 2020 [3]. Therefore, it is of paramount importance to the public that cellular and molecular mechanisms, which can be targeted in order to prevent and ultimately treat depression, must be identified.

Various studies and research have verified that over-production of cytokines is the key factor responsible for neurological inflammation, and this phenomenon is essential with regard to the psychopathology of depression [4–6]. Nonetheless, there is little knowledge regarding the molecular mechanisms behind the relationship and correlation between depression and central nervous system (CNS) inflammation. Interleukin-1 beta (IL-1β) is one of the many important pro-inflammatory cytokines and has been proven to participate in the inflammatory responses that take place in the brain. These inflammatory responses eventually lead to cellular damage in stress-relevant neuropsychiatric diseases, such as co-inherited MDD [7]. Recent research has suggested that both stress and IL-1β are responsible for the decline in hippocampal neurogenesis, which is, in turn, associated with the development of depressive-like behavior [8]. A meta-analysis demonstrated that antidepressant therapies resulted in a reduction in IL-1β serum level and simultaneously alleviated depressive symptoms [9]. This phenomenon suggests that IL-1β plays a key role in the pathogenesis of depression. NOD-like receptor protein 3 (NLRP3), the intracellular pattern recognition receptor, is composed of two components—the apoptosis-associated speck-like protein containing a CARD (ASC) and the effector protein caspase-1 [10]. This multi-protein platform formation leads to the autocatalysis and activation of caspase-1, which, in turn, converts IL-1β and IL-18 into their respective biological active forms, prior to secretion. Recently, there has been evidence that suggests inflammasome and IL-1β activities might contribute to the development of several common human diseases, which include gout, type 2 diabetes, non-alcoholic steatohepatitis, atherosclerosis, Alzheimer’s disease, and cancer [11, 12]. Moreover, various novel studies implied that the NLRP3 inflammasome is involved in stress-induced depression and, therefore, may be a potential target for the treatment of depression [13].

Evidence also suggests that depression is likely to be a type of microglial disease [14]. Microglia make up approximately 10% of the entire brain, and they are the main line of innate immune defense in the brain [15]. In addition, they contribute to the normal development and regulation of ongoing structural and functional processes of individual synapses to neural circuits to behavior [16, 17]. Microglial cells are activated during pathological conditions, such as infection, injury, and neurodegeneration, to orchestrate and execute various inflammatory, protective, recuperative, and toxic processes, which can have a detrimental impact on brain cells, including neurons and glial cells. Owing to the responsiveness of microglia towards those pathological conditions, they are, therefore, regarded as important diagnostic markers of inflammation onset or CNS disease progression [18]. Specific targets such as microglial cell surface and mitochondrial receptors have been utilized to develop neuro-inflammation diagnostic biomarkers. An 18-kDa translocator protein (TSPO), a protein situated in the outer mitochondrial membrane, is an example of a widely applicable diagnostic target for neuro-inflammation. It is specifically upregulated in activated microglia during brain injuries and neurodegenerative diseases that are correlated to neuro-inflammation [19, 20]. Various TSPO ligands are labeled using radioisotopes for autoradiography or in vivo neuro-inflammatory PET scan purposes [21, 22]. [18F] DPA-714 is advantageous in comparison to other radioligands of TSPO, with regard to high binding affinity and target-to-background ratio. Furthermore, F-18’s physical property (− *t*1/2 = 109.8 min) makes it a very appropriate candidate for in vivo PET imaging [23].

In this research, our aim is to evaluate microglial activation and hippocampal neuro-inflammation in a chronic mild stress (CMS)-induced depression model, by using a combinatory approach of in vivo [18F] DPA-714 PET imaging, ionized calcium-binding adapter molecule 1 (Iba-1) and TSPO immunohistochemistry, and detection of NLRP3 inflammasome and some inflammatory mediators. We also observed the effect of minocycline on hippocampal neuro-inflammation and depressive-like behavior induced by chronic mild stress. We place strong emphasis on the implementation of the CMS-induced depression model so as to explore the potential role of microglial activation in CMS-induced depression.

## Methods

### Experimental design

To facilitate the assessment of the impact of chronic mild stress on neuro-inflammation and behavioral parameters as well as the efficacy of the minocycline, the current study consisted of two experiments, both of which used the same CMS protocol.

### Experiment 1: change of microglia, NLRP3 inflammasome, and cytokines in the hippocampus of rats submitted to CMS

**Fig. 1:**
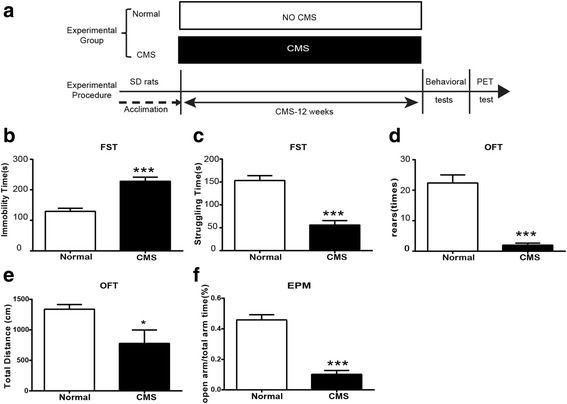
CMS induces depressive-like behavior in rats. **a** The experimental paradigm. **b** Time spent immobile in the FST. **c** Time spent struggling in the FST. **d** Rearing numbers in the OFT. **e** Total distance traveled in the OFT. **f** Open-arm time percent in the EPM test (*n* = 8/group). All data are expressed as the mean ± SEM. *p < 0.05, ***p < 0.001, compared to normal rats

The rats were randomly divided into two groups. The first group was the normal control; another group was exposed to CMS for 12 weeks. Twenty-four hours after the last stressor, three rats of each group were randomly selected to receive PET measurement. Other rats of each group were sacrificed after behavior test from which we collected brain tissue (please see Fig. 1a).

### Experiment 2: efficacy of the minocycline on hippocampal neuro-inflammation and depressive-like behavior induced by CMS

As illustrated in Fig. 6a, the rats were randomly assigned into four groups. The first group was the normal control; the remaining three groups were exposed to 12-week CMS, among which, two groups were treated simultaneously with either saline or minocycline (i.p., 50 mg/kg, diluted in saline) for 4 weeks (weeks 9–12 of CMS). Twenty-four hours after the last stressor, rats of each group received behavior test in sequence and then sacrificed. And, brain tissue was collected.

### Animals

The experiments were carried out on male Sprague Dawley (SD) rats (6–7 weeks old) weighting between 180 and 200 g, obtained from the Shanghai Laboratory Animal Center of China, where the rats were housed in groups after born. Rats were housed in groups of two to four per cage under a 12-h light/dark cycle with access to food and water ad libitum (except when indicated). The stress intervention began after a 1-week habituation period. The behaviors test was conducted after 12-week CMS when the rats were 19–20 weeks old. This study was carried out in accordance with the National Institutes of Health Guide for the Care and Use of Laboratory Animals. The protocol was approved by the Animal Ethics Committee of Shanghai Medical College, Fudan University, Shanghai, China (20120302-107).

### CMS procedure

CMS is a rodent model of depression in which animals are exposed to a random sequence of mild stressors. Rats were subjected to nine different stressors: water deprivation (24 h), food deprivation (24 h), light/dark cycle reversal, hot environment (40 °C, 5 min), swimming in cold water (4 °C, 5 min), cage shake (15 min), restraint (2 h), radio noise in the room (12 h), and flashing light (12 h). These stressors were performed once per day in a random order for 12 weeks.

### Behavioral testing

Twenty-four hours after the end of CMS, the open field test (OFT), the elevated plus maze (EPM), and the forced swim test (FST) were conducted in sequence (test/day).

The OFT was performed as previously described [23]. Rats were placed in the center of a Plexiglas box (100 cm × 100 cm × 40 cm) in a brightly lit room. During a 5-min session, animals were scored for the number of rearing behaviors and the distance traveled in the box. Animal behavior was recorded and subsequently analyzed using a video-tracking system (Shanghai Mobile Datum Information Technology Company, Shanghai, China).

The EPM was shaped like a plus sign and consisted of a central platform (5 × 5 cm), two opposite open arms (30 cm × 5 cm), and two equal-sized opposite closed arms, elevated 50 cm from floor and illuminated by a dim light. Individual trials lasted for 5 min each and were recorded with a video-tracking system (Shanghai Mobile Datum Information Technology Company, Shanghai, China). Open-arm time percentage ([time in open arms] / [time in total arms] × 100) was calculated as described in our previous paper [24]. All behavioral tests were performed by an individual who was blind to the animal’s treatment status.

In the FST, rats were individually put into an 18- or 15-cm-diameter glass cylinder filled to 30 cm with 23 ± 1 °C water. During analysis of the recordings, immobility was defined as the absence of all movement except motions required to maintain the animal’s head above the water. Struggling was defined as vigorous movements with forepaws breaking the water. Results were expressed as time (in seconds) that animals spent immobile or struggling during a 5-min session.

### [18F] DPA-714 PET

Small-animal PET was performed with a micro-PET scanner (Siemens Inc.). Under isoflurane anesthesia, the rats were placed prone in the center of the field of view of the scanner and injected with [18F] DPA-714 via the lateral tail vein. Whole-brain scanning was performed after radiotracer injection, and 10-min static PET images were acquired. The quantification analysis of PET images was performed using the same method as previously reported [25].

### Minocycline treatment

Minocycline (Sigma-Aldrich) was used as a microglial activation inhibitor to examine whether CMS-induced microglial activation is involved in the development of depressive-like behavior and the underlying mechanisms. Rats were intraperitoneally injected of saline and minocycline (50 mg/kg, diluted in saline) once daily during the last 4 weeks of 12-week CMS (weeks 9–12 of CMS).

### Immunohistochemical analysis

The brains were separated and post-fixed in 4% PFA at 4 °C overnight and immersed in 20% sucrose (4% PFA as solvent) followed by 30% sucrose (in 0.1 M PBS). The brain samples were cut into 30-μm-thick sections (CM1850; Leica Microsystems, Wetzlar, Germany). Sections were incubated in 0.01 mol/L citrate buffer (pH 6.0) for high-temperature antigen retrieval. Tissues were blocked in 2% (*w*/*v*) BSA (Sigma) and then exposed overnight to the following primary antibody mixtures: anti-Iba-1 (1:500, Abcam), anti-PBR (1:500, Santa Cruz), and anti-NLRP3 (1:500, Abcam) at 4 °C. Detection of primary antibodies was performed with secondary antibodies (donkey anti-rabbit, Alexa 594 conjugated, 1:1000, Invitrogen, USA; donkey anti-goat, Alexa 594 conjugated, 1:1000, Invitrogen, USA; donkey anti-goat, Alexa 488 conjugated, 1:1000, Invitrogen, USA; donkey anti-rabbit, Alexa 488 conjugated, 1:1000, Invitrogen, USA; Hoechst, 1:1000, Beyotime, China) for 1 h in the dark. The sections were then washed five times with PBS in the dark. Immunofluorescence sections were observed with a Leica SP5 fluorescence microscope, using excitation wavelengths of 633 nm (helium/neon2, blue Cy5 labeling), 543 nm (helium/neon1, red Cy3 immunofluorescence), and 488 nm (argon, yellow green Cy2 immunofluorescence), and images were captured with a CCD spot camera for data analysis. The same region per hippocampal section and three sections per animal were counted by experimenters who were blind to the experiment design.

### Western blot analysis

The rats’ hippocampi were homogenized in RIPA buffer (Thermo Scientific) with protease inhibitors (Beyotime). Protein samples were run on 12% Tris-glycine SDS-PAGE gels, transferred to PVDF membrane (0.2 or 0.45 μm), and blotted with antibodies against Iba-1 (1:1000, Abcam), CD11b (1:1000, Abcam), NLRP3 (1:1000, Abcam), caspase-1 (1:1000, Abcam), ASC (1:200, Santa Cruz), IL-1β (1:1000, R&D System), GAPDH (1:20,000), and β-actin (1:20,000). Primary antibody incubation was performed overnight at 4 °C. Secondary antibodies (1:10,000, EarthOx) were incubated for 2 h at room temperature. The signal was captured on an ImageQuant LAS4000 mini image analyzer (GE Healthcare, Buckinghamshire, UK), and the band levels were quantified using ImageJ software (NIH, Bethesda, MD, USA).

### Quantitative real-time RT-PCR

Total RNA was isolated from the rats’ hippocampi using TRIzol reagent (Invitrogen) according to the manufacturer’s instructions. cDNA was synthesized by a Prime Script Kit (Bio-Rad). Quantitative real-time PCR was performed by using gene-specific primers and SYBR Premix Ex Taq (Bio-Rad, CA, USA). Oligonucleotide primers specific for rat are IL-1β (F 5′ TTCTTTGAGGCTGACAGACC 3′; R 5′ CGTCTTTCATCACACAGGAC 3′), NLRP3 (F 5′ AGTGGATAGGTTTGCTGGGATA 3′; R 5′ CTGGGTGTAGCGTCTGTTGAG 3′), ASC (F 5′ GAAGAGTCTGGAGCTGTGG 3′; R 5′ AATGAGTGCTTGCCTGTG 3′), caspase-1 (F 5′ AGTGTAGGGACAATAAATGG 3′; R 5′ GATGGACCTGACTGAAGC 3′), IL-6 (F 5′ ACTTCCAGCCAGTTGCCTTCTTG 3′; R 5′ GGTCTGTTGTGGGTGGTATCCTC 3′), iNOS (F 5′ GCACAGAGGGCTCAAAGG 3′; R 5′ CACATCGCCACAAACATAAA 3′), IL-4 (F 5′ GAACCAGGTCACAGAAAAAGGGA 3′; R 5′ TGGGAAGTAAAATTTGCGAAGCA 3′), IL-10 (F 5′ TGCCTTCAGCAGAGTGAAG 3′; R 5′ GGGAAGAAATCGATGACAG 3′), and GAPDH (F 5′ CCCTTCATTGACCTCAACTAC 3′; R 5′ CTTCTCCA TGGTGGTGAAGAC 3′). Relative messenger RNA (mRNA) expression levels were analyzed using the formula 2^−ΔΔCt^ method and normalized to the GAPDH ribosomal RNA.

### Statistical analysis

All data were analyzed using SPSS 16.0 (SPSS Inc., Chicago, USA). The data collection and analysis were performed independently by two experimenters. Results are expressed as the mean ± standard error of the mean. Data were analyzed using one- or two-way analysis of variance (ANOVA) according to the factors introduced in the experimental design. Where F ratios were significant, post hoc comparisons were made using Tukey’s post hoc test. Significance levels were set at *p* < 0.05.

## Results

### CMS induces depressive-like behavior in rats

To clarify the role of neuro-inflammation in depression, we subjected SD rats to the CMS protocol (Fig. 1a). This protocol involves multiple, daily, mild stressors at unpredictable times proven to induce depressive- and anxiety-like behavior. As expected, after 12 weeks of CMS, stressed rats displayed depressive-like behavior as shown by increased immobility time and decreased struggling time in the FST (a measure of despair with good predictive value for antidepressant effects; Fig. 1b, c) and reduced rearing numbers (a measure of exploration; Fig. 1d) in the OFT, when compared to non-stressed control animals. As shown in Fig. 1e, CMS also reduced the locomotor activity of rats as shown by decreased total distance in the OFT. Additionally, stressed rats showed a significant decrease in the time spent in the open arms of EPM as compared to non-stressed control animals (Fig. 1f). These results indicate that CMS successfully induced depressive- and anxiety-like behavior.

### Multi-modal detection of hippocampal microglial activation in the rats exposed to CMS

**Fig. 2:**
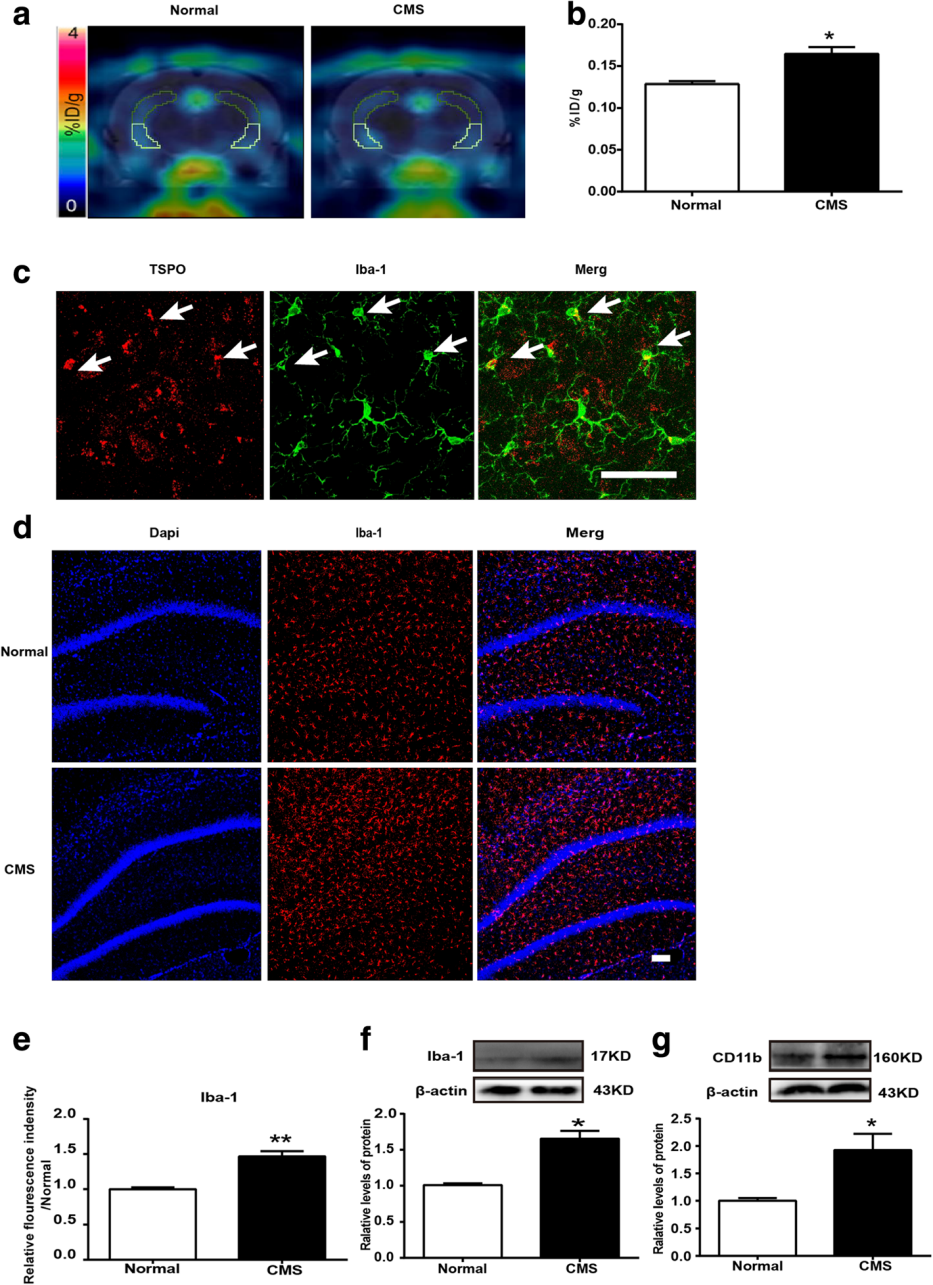
Multi-modal detection of hippocampal neuro-inflammation in the rats exposed to CMS. **a** The representative PET images. **b** DPA-714 accumulation (*n* = 3/group). **c** Immunofluorescence staining of hippocampal sections from normal rats. TSPO (red), Iba-1 (green); scale bar, 50 μm. Arrows indicate the cells positive for Iba-1 and TSPO. **d** Iba-1/DAPI (red/blue) staining in the hippocampus; scale bar, 100 μm. **e** Qualification of Iba-1 immunofluorescence density (*n* = 4/group). **f** Western blot analysis of Iba-1 (*n* = 4/group). **g** Western blot analysis of CD11b (*n* = 4/group). All data are expressed as the mean ± SEM. *p < 0.05, **p < 0.01, compared to normal rats

Recently, neuro-inflammation, characterized in particular by microglial activation, has been suggested as a possible mechanism of stress-induced depressive disorders. An experimental radiopharmaceutical specific of TSPO ligand, namely [18F] DPA-714, allows quantifying this microglial activation using PET imaging. To examine the effect of chronic mild stress on TSPO inflammatory signals, [18F] DPA-714 uptake was compared at 12-week treatment time points. As illustrated in Fig. 2a, accumulation of [18F] DPA-714 in the hippocampus was greater in stressed rats than in non-stressed control rats. The brain uptake of [18F] DPA-714 expressed as %ID/g was calculated (Fig. 2b). The results exhibited that CMS significantly increased [18F] DPA-714 signals at the hippocampus.

Then, we also detected the expression of TSPO in the hippocampus of normal rats using the immunohistochemical technique. The results showed that TSPO was predominantly expressed by microglia (Fig. 2c) and further confirmed that [18F] DPA-714 signal correlated well with microglial activation. We also observed the amount of Iba-1-labeled microglial cells was significantly larger in the hippocampus of stressed rats than in non-stressed control rats (Fig. 2d, e). We then analyzed protein levels of Iba-1 and another microglial marker protein CD11b by western blotting. The results showed that protein levels of Iba-1 (Fig. 2f) and CD11b (Fig. 2g) increased significantly in the hippocampus of stressed rats compared with non-stressed control rats. Briefly, immunohistochemical analysis and western blot assay further confirmed PET imaging results and indicated chronic mild stress induced microglial activation in the hippocampus.

### CMS increases gene expression of inflammatory factors in the hippocampus

**Fig. 3:**
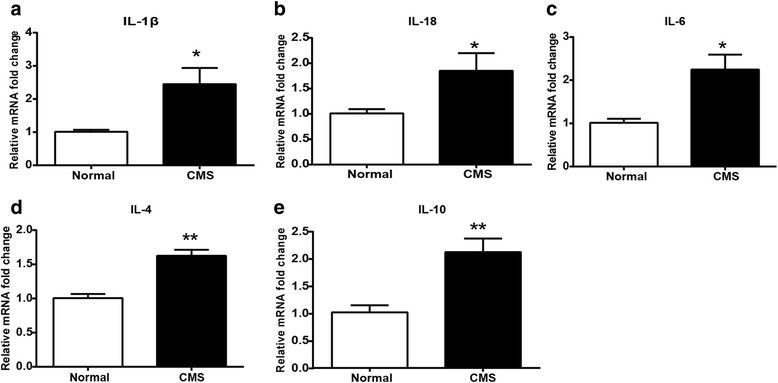
Detection of cytokine changes in the hippocampus of CMS rats. **a**–**e** Real-time PCR analysis of IL-1β, IL-18, IL-6, IL-4, and IL-10 (*n* = 4–7/group). All data are expressed as the mean ± SEM. *p < 0.05, **p < 0.01, compared to normal rats

To further study the putative changes induced by chronic stress on hippocampal milieu, we measured the hippocampal inflammatory gene expression by qPCR. As shown in Fig. 3a–e, chronic stress not only increased IL-1β, IL-18, and IL-6 gene expression but also enhanced the mRNA levels of anti-inflammatory cytokines, such as IL-4 and IL-10, in the hippocampus of rats. These results confirmed that chronic stress induced the hippocampal neuro-inflammation, which suggested by the upregulation of inflammatory mediators in the hippocampus.

### CMS causes the cleavage of IL-1β and caspase-1

**Fig. 4:**
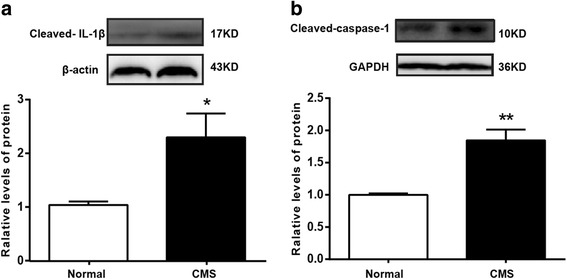
CMS causes the cleavage of IL-1β and caspase-1. **a**, **b** Western blot analysis of cleaved IL-1β and cleaved caspase-1 (*n* = 4/group). All data are expressed as the mean ± SEM. *p < 0.05, **p < 0.01, compared to normal rats

There is some evidence that IL-1β is an important mediator of stress-induced depressive-like behavior and depression. As shown above, the hippocampal IL-1β mRNA was upregulated in stressed rats. Then, is the active form of IL-1β also increased? The results exhibited that the mature form of IL-1β and its convertase, cleaved caspase-1, were both increased in the hippocampus after exposure to chronic stress (Fig. 4a, b). These results suggest that chronic stress induced caspase-1 activation and IL-1β maturation in the hippocampus of rats.

### Changes of NLRP3 inflammasome in the hippocampus of CMS rats

**Fig. 5:**
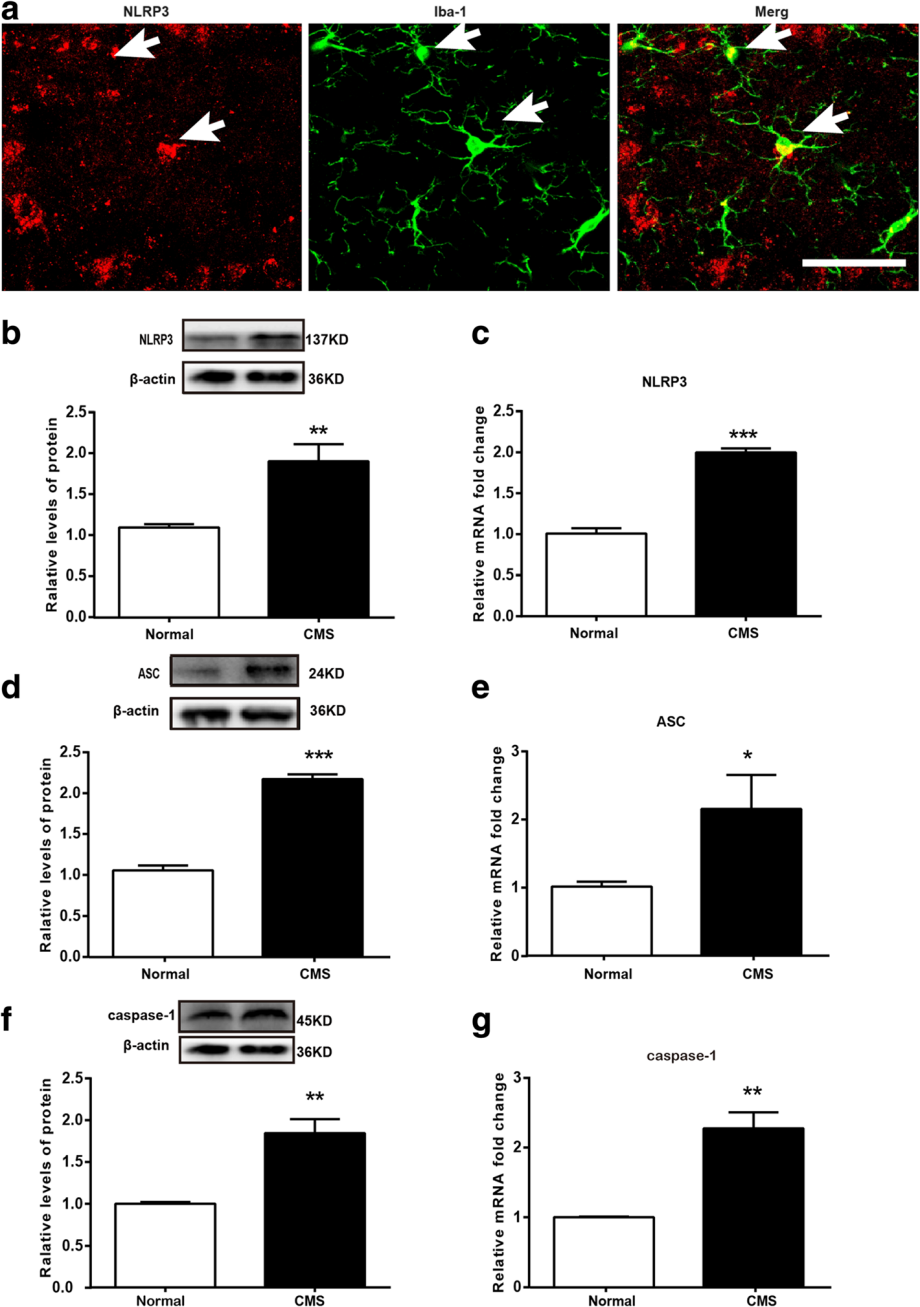
Changes of NOD-like receptor protein 3 (NLRP3) inflammasome in the hippocampus of CMS rats. **a** Immunofluorescence staining of hippocampal sections from normal rats. NLRP3 (red), Iba-1 (green); scale bar, 100 μm. Arrows indicate the cells positive for Iba-1 and NLRP3. **b** Western blot analysis of NLRP3. **c** PCR analysis of NLRP3. **d** Western blot analysis of ASC. **e** PCR analysis of ASC. **f** Western blot analysis of caspase-1 **g** PCR analysis of caspase-1 (*n* = 4–7/group). All data are expressed as the mean ± SEM. *p < 0.05, **p < 0.01, ***p < 0.001, compared to normal rats

As we know, the maturation of IL-1β, that is to say the cleavage of caspase-1, is dependent on NLRP3 inflammasome. We then further detected the expression of NLRP3 in the hippocampus of normal rats. The results showed that NLRP3 was predominantly expressed by microglia (Fig. 5a) and the three components of the NLRP3 inflammasome, including NLRP3 (Fig. 5b, c), ASC (Fig. 5d, e), and caspase-1 (Fig. 5f, g), displayed significantly higher protein and mRNA expression levels in the rats exposed to CMS compared to the control group. All these data hint that the NLPR3 inflammasome has a vital role in CMS-induced depressive-like behavior.

### Chronic minocycline treatment reversed CMS-induced depressive-like behavior

**Fig. 6:**
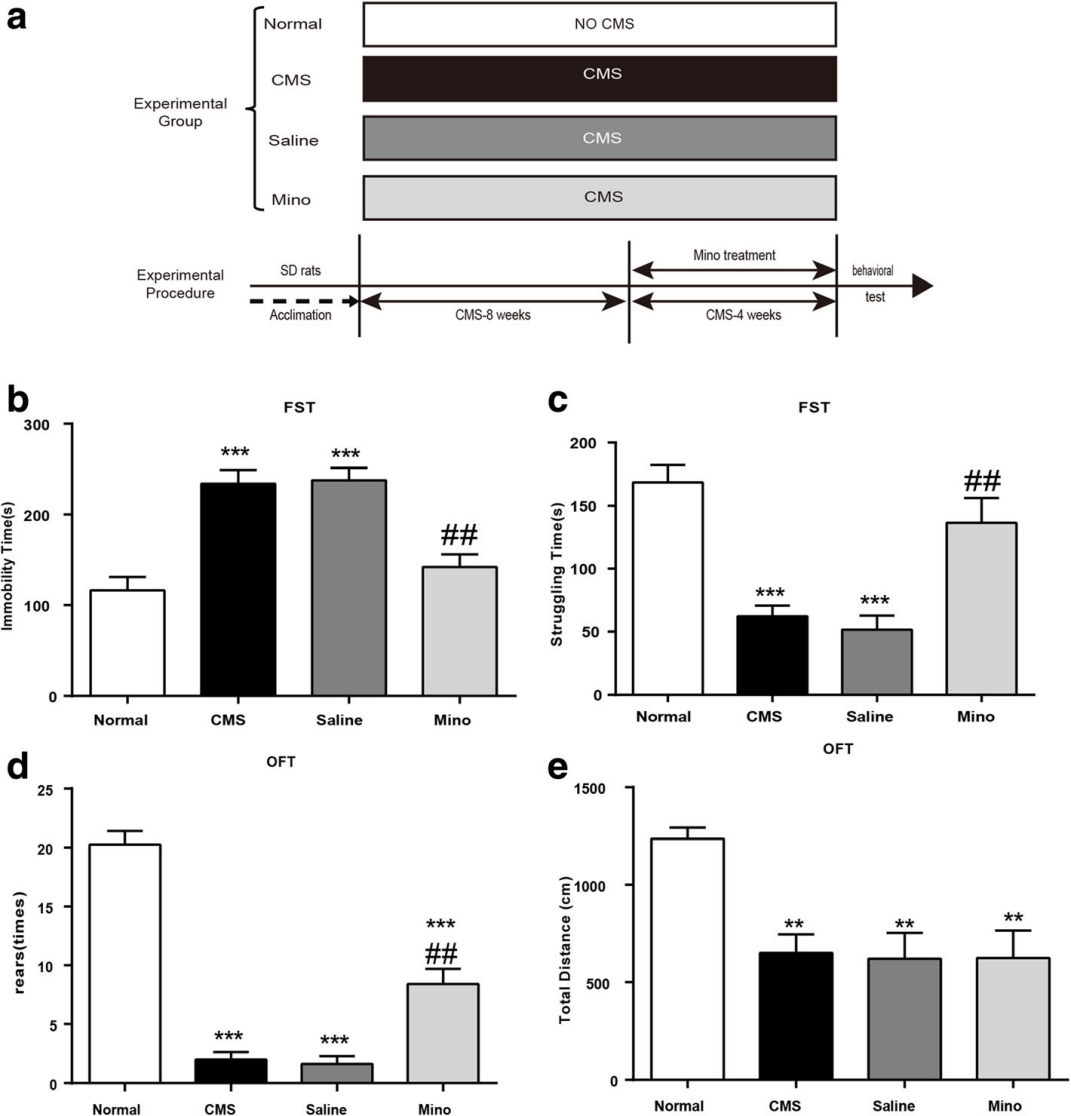
Chronic minocycline treatment reversed CMS-induced depressive-like behavior. **a** Experimental paradigm. **b** Immobility time in the FST. **c** Struggling time in the FST. **d** Rearing numbers in the OFT. **e** Total distance traveled in the OFT (*n* = 5–8/group). All data are expressed as the mean ± SEM. ^##^*p* < 0.01, compared to saline-treated rats. **p < 0.01, ***p < 0.001, compared to normal rats

Minocycline, as a common inhibitor of microglial activation, can be used to assess the role of microglial activation in stress-induced depressive-like behavior. Chronic treatment with minocycline (animal treatment paradigm exhibited in Fig. 6a) can significantly normalize the behavioral deficits of stressed rats, indicated by spending less time immobile and more time struggling (Fig. 6b, c). In the OFT, minocycline treatment increased the number of rearing but still has a significant difference from the normal group. Meanwhile, minocycline treatment had no effect on the total distance moved (Fig. 6d, e).

### Chronic minocycline treatment dampened microglial activation, NLRP3 inflammasome, and pro-inflammatory mediators in rats exposed to CMS

**Fig. 7:**
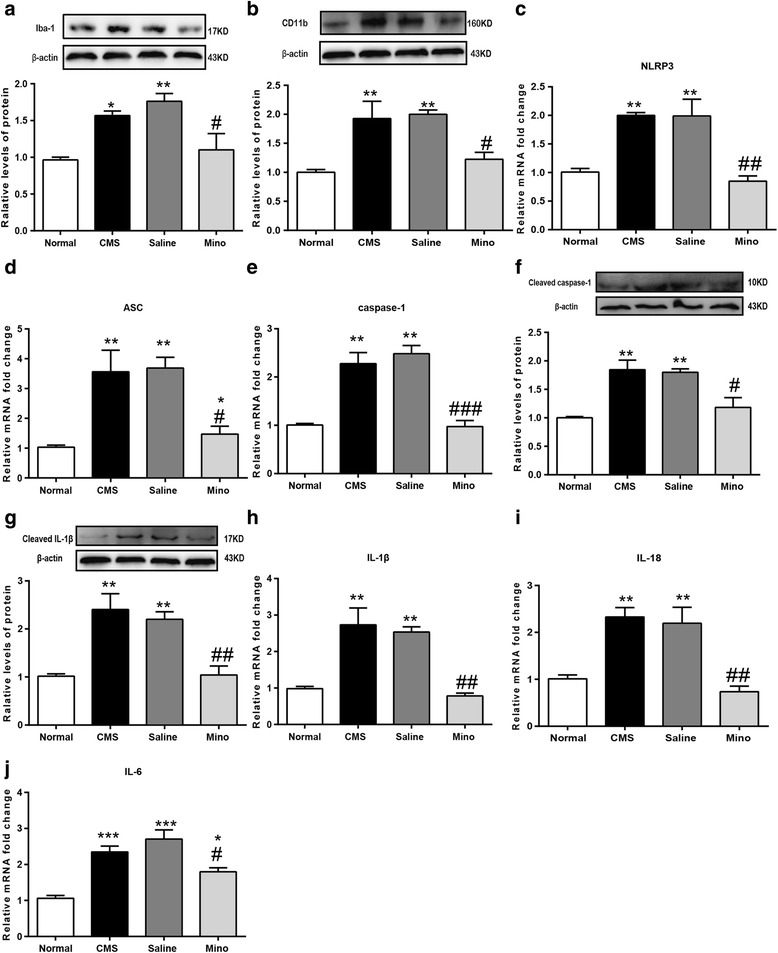
Chronic minocycline treatment dampened microglial activation, NLRP3 inflammasome, and pro-inflammatory mediators in rats exposed to CMS. **a** Western blot analysis of Iba-1 (*n* = 4/group). **b** Western blot analysis of CD11b (*n* = 4/group). **c**–**e** PCR analysis of NLRP3, ASC, and caspase-1 (*n* = 4/group). **f**, **g** Western blot analysis of cleaved caspase-1 and cleaved IL-1β (*n* = 4/group). **h**–**j** PCR analysis of IL-1β, IL-18, and IL-6 (*n* = 4/group). All data are expressed as the mean ± SEM. ^#^*p* < 0.05, ^##^*p* < 0.01, ^###^*p* < 0.001, compared to saline-treated rats. *p < 0.05, **p < 0.01, ***p < 0.001, compared to normal rats

The effect of minocycline on microglial activation was checked by examining the protein of the microglial cell surface markers Iba-1 and CD11b. Data revealed that chronic minocycline intake significantly normalized Iba-1 and CD11b expression in the hippocampus of CMS rats (Fig. 7a, b). Furthermore, we found that the abovementioned antidepressant effect of minocycline maybe was associated with decreased NLRP3 inflammasome activity. The minocycline treatment significantly inhibited CMS-induced NLRP3 inflammasome component gene expression (NLRP3, ASC, and pro-caspase-1) (Fig. 7c–e), among which, ASC gene expression still remained significantly different between minocycline-treated and normal subjects. Meanwhile, after minocycline treatment, matured caspase-1 and IL-1β were significant normalized (Fig. 7f, g). Besides, chronic minocycline treatment also remarkably prevented the increase of mRNA levels of the pro-inflammatory cytokines, such as IL-1β, IL-18, and IL-6. But, IL-6 mRNA is still higher in minocycline-treated subjects as compared with normal subjects (Fig. 7h–j).

## Discussion

In our present study, we detected hippocampal neuro-inflammation by implementing PET imaging with the specific TSPO ligand—[18F] DPA-714 in a CMS rat model of depression. We were able to correctly identify that activated microglia are the possible cellular correlate of increased [18F] DPA-714 uptake thanks to the combination of PET imaging and ex vivo TSPO and Iba-1 antibody staining on the brain sections. We also detected an increase of NLRP3 inflammasome and some inflammatory mediators in the hippocampus after chronic stress and found that minocycline treatment can alleviate the depressive- and anxiety-like behavior and neuro-inflammation induced by chronic stress. This study highlighted that microglial activation is very likely to play a role in CMS-induced depressive- and anxiety-like behavior via NLRP3 inflammasome-IL-1β signaling.

The CMS procedure utilized in this study is a validated animal model that represents the pathophysiology of human depression [26]. In our hypothesis, we suggested that CMS is responsible for a myriad of complex human depression symptoms, which include physiological change increment [27], hopelessness state augmentation, and motivation loss [28]. Hence, this model is frequently utilized to analyze the underlying cellular and molecular mechanisms behind the pathophysiology of depression. We confirmed that rats that are exposed to CMS for 12 weeks became more immobile and struggled less during the FST, exhibited a decline in rearing behaviors during OFT, and spent less time in the open arms during the EPM test. These characteristics are consistent with our previous reports [24].

We hypothesized that [18F] DPA-714 PET imaging, which is a non-invasive marker of microglial cell activation, is very likely to be beneficial with regard to detecting and following immunological status of the brains in rat models of depression. By using [18F] DPA-714 PET imaging of TSPO, it is evident that the amount of [18F] DPA-714 that has successfully bound to the hippocampus of CMS rats was increased in comparison to that of control animals in our study. Consistent with these findings, a recent study observed an increase in brain TSPO total distribution volume (VT), an index of TSPO density, in patients with MDD compared with healthy and age-matched control participants using [18F] FEPPA PET [29]. However, to present, there has been no study available about the neuro-inflammatory changes in rats using PET imaging in the depression model. However, it is an encouraging sign that specific TSPO ligands have been investigated as potential therapies for neurological disorders such as Alzheimer’s disease [30], multiple sclerosis [31], neuropathic pain [32], peripheral nerve injury [33], and anxiety disorders [34]. Several TSPO ligands are labeled using radioisotopes to detect neuro-inflammation using either autoradiography or in vivo PET imaging. Among them, [18F] DPA-714 is advantageous with regard to possessing high binding affinity and target-to-background ratio in comparison to other TSPO radioligands. A pilot clinical study confirmed the in vivo stability and biodistribution, and acceptable effective dose estimation of [18F] DPA-714 in healthy humans, which supports further clinical trials and preclinical evaluation with PET imaging [35]. Therefore, in our present study, we first utilized the [18F] DPA-714, the PET ligand which binds to active-stage TSPO that is upregulated on microglia, to demonstrate neuro-inflammation imaging in rat models of depression.

A few studies addressed that PET imaging of TSPO (18 kDa) can be utilized to detect activated microglial cells in the brain [36, 37]. TSPO expressivity is low in healthy brains. However, it is elevated in diseases that are correlated to neuro-inflammation such as stroke, trauma, infection, and auto-immune and neurodegenerative disorders [38, 39]. Nevertheless, it is crucial to bear in mind that TSPO can also be expressed by reactive astrocytes [40]. In order to demonstrate this, we showed that TSPO is situated within the microglia in the rat hippocampal region by performing IHC double-staining using TSPO antibody and Iba-1 antibody. Both were capable of corroborating with [18F] DPA-714 PET imaging in rats. An increase in TSPO radiotracer binding, which was detected by PET, is likely to be attributed to microglial activation. Hence, we deduced that an increase in TSPO binding is evidence of neuro-inflammation. Coupled with recent evidence regarding the targeting of TSPO, this may provide new therapeutic opportunities. Our present study will prompt a strong interest on both the preclinical and clinical TSPO in vivo imaging for depressive disorders.

Neuro-inflammation is defined as the cellular and biochemical responses to numerous insults that occur within the central nervous system, which include the activation of microglia, also known as the macrophages of the CNS. Microglia play an important role by acting as an innate immune response to invading pathogens and by initiating adaptive responses via antigen presentation [41]. In this study, we explored the expression of microglial activation biomarkers at the end of the 12-week CMS procedure. In our model, we discovered that Iba-1 and CD11b are both significantly over-expressed in the CMS-exposed rats’ hippocampi, which implied that microglia are activated in CMS-exposed rats, and activated microglia possess hyper-ramified or amoeboid/phagocytic properties. Hyper-ramified microglial morphology composed of increased arborization with thick processes; however, these processes were retracted and the cell bodies became enlarged when transformation of hyper-ramified microglia into amoeboid form commences. Activated microglia produce pro-inflammatory cytokines such as IL-1β, tumor necrosis factor (TNF), and IL-6, just to highlight a few. We also detected pro-inflammatory mediator—IL-1β, IL-18, and IL-6—gene expressivity in the hippocampus. There are significant increases in the rats exposed to CMS as compared to that of the control group. Preclinical and clinical research studies that establish relationships and links between psychiatric illnesses and inflammatory processes have burgeoned over the past two decades. This is attributable to an attempt to link illnesses, especially MDD, with “stress” biology, which increases the possibility of the existence of an “initial common pathway” whereupon the combinatory effects of immune/inflammatory and stress biomarkers are capable of producing changes in both brain structure and functions.

IL-1β might mark the start of the pro-inflammatory responses targeted specifically at psychological stress by resulting in a cascade of inflammatory cytokine responses [13]. It has been speculated that depression is related to an increased secretion of cytokines, in particular IL-1β, by macrophages [42]. Koo and Duman discovered that depressive behavior and cellular responses, which are attributable to chronic stress exposure, can be halted by IL-1β blockade [43]. In our research, IL-1β protein and mRNA expression were both markedly increased in the brains of the CMS group rats. This finding was compatible with several studies in patients with depression, and animal models [44, 45]. Our data also demonstrated that IL-1β might play a key role in CMS-induced depressive-like behavior in rats.

IL-1β precursor needs to be biologically activated via proteolytic cleavage prior to secretion [46]. Caspase-1, which is also known as an IL-1β-converting enzyme, is responsible for IL-1β maturation by cleaving the 31-kDa IL-1β precursor into 17-kDa biologically active forms [47]. The significant increase in active IL-1β forms and IL-1β mRNA in the hippocampus of CMS implied that there is a possibility for caspase-1 to be activated in the brain, which was justified by the increase of active caspase-1 form (p10). It is well known that many types of stimuli, such as bacterial compounds, viruses, and endogenous molecules released from injured cells, activate caspase-1 via NLRP3 inflammasome—a crucial component in caspase-1 activation [48–50]. We postulated that there were higher expression levels of NLRP3 inflammasome components. As predicted, all three components of the NLRP3 inflammasome had significantly higher mRNA and protein expression levels, when measured by real-time RT-PCR and western blotting, respectively, in the CMS-exposed rats’ brains compared with those of the control rats, including NLRP3, ASC, and caspase-1. These data illustrated that the NLPR3 inflammasome might play a central role in the rats’ CMS-induced depressive-like behavior.

To characterize further the role of microglia in the observed CMS-induced depressive-like behavior, we used minocycline, which has been shown to have several biological effects including inhibition of cytochrome C release from mitochondria, inhibition of caspase expression, and suppression of microglial activation [51]. Different timing and duration of minocycline treatment were used according to different stress procedures. Some studies got the similar results in which minocycline was given concurrently with chronic exposure [52, 53] or following the stressor intervention [54]. In our study, minocycline treatment was given during the last period of CMS to evaluate its antidepressant effect. We found that minocycline administration completely inhibited the increase and activation of microglia in the hippocampus and reduced CMS-induced depressive-like behaviors in the FST and OFT. Moreover, minocycline administration also inhibited expression of pro-inflammatory factors and reduced CMS-induced increased levels of the components of NLRP3 inflammasome in the hippocampus, which indicates that neuro-inflammation induced by microglia may play a vital role in the pathogenesis of CMS-induced depressive-like behavior. This is consistent with previous studies using animal models of depression [54]. Thus, we inferred that microglial activation indeed plays a pivotal role in the regulation of CMS-induced depressive-like behaviors.

## Conclusion

Our present results suggest that [18F] DPA-714 is a suitable PET ligand for imaging in depression model rats. The increase in TSPO in all these pathologies is mirrored by an analogous behavior in the main corresponding preclinical models, thereby supporting the use of TSPO tracers for the study of microglial activation and TSPO upregulation, despite the existence of some differences, related to different tracers, which are emerging in similar cases. Coupled to recent evidences regarding the targeting of TSPO may provide new therapeutic opportunities. Our present study will prompt a strong interest on both the preclinical and clinical TSPO in vivo imaging for depressive disorders. Furthermore, the present study suggests that the NLRP3 inflammasome is involved in CMS-induced depressive-like behavior in rats. The NLRP3 inflammasome may be a central mediator between immune activation and development of depression. It raises the possibility that the NLRP3 inflammasome can be a more specific target for the development of novel pharmacological agents for depression treatments in the near future.

## Abbreviations

ASC: Apoptosis-associated speck-like protein containing a CARD
CMS: Chronic mild stress
CNS: Central nervous system
EPM: Elevated plus maze
FST: Forced swim test
Iba-1: Ionized calcium-binding adapter molecule 1
IL-1β: Interleukin-1β
MDD: Major depressive disorder
NLRP3: NOD-like receptor protein 3
OFT: Open field test
PET: Positron emission computed tomography
SD: Sprague Dawley
TNF: Tumor necrosis factor
TSPO: Translocator protein

## References

[1] Depression. http://www.who.int/mediacentre/factsheets/fs369/en/. Updated February 2017.

[2] Kessler RC, Berglund P, Demler O, Jin R, Koretz D, Merikangas KR, Rush AJ, Walters EE, Wang PS, National Comorbidity Survey R (2003). The epidemiology of major depressive disorder: results from the National Comorbidity Survey Replication (NCS-R). JAMA.

[3] Ustun TB, Ayuso-Mateos JL, Chatterji S, Mathers C, Murray CJ (2004). Global burden of depressive disorders in the year 2000. Br J Psychiatry.

[4] Maes M, Leonard B, Fernandez A, Kubera M, Nowak G, Veerhuis R, Gardner A, Ruckoanich P, Geffard M, Altamura C (2011). (Neuro)inflammation and neuroprogression as new pathways and drug targets in depression: from antioxidants to kinase inhibitors. Prog Neuro-Psychopharmacol Biol Psychiatry.

[5] Maes M (1995). Evidence for an immune response in major depression: a review and hypothesis. Prog Neuro-Psychopharmacol Biol Psychiatry.

[6] Dowlati Y, Herrmann N, Swardfager W, Liu H, Sham L, Reim EK, Lanctot KL (2010). A meta-analysis of cytokines in major depression. Biol Psychiatry.

[7] Gadek-Michalska A, Tadeusz J, Rachwalska P, Bugajski J (2013). Cytokines, prostaglandins and nitric oxide in the regulation of stress-response systems. Pharmacol Rep.

[8] Chesnokova V, Pechnick RN, Wawrowsky K (2016). Chronic peripheral inflammation, hippocampal neurogenesis, and behavior. Brain Behav Immun.

[9] Hannestad J, DellaGioia N, Bloch M (2011). The effect of antidepressant medication treatment on serum levels of inflammatory cytokines: a meta-analysis. Neuropsychopharmacology.

[10] Petrilli V, Dostert C, Muruve DA, Tschopp J (2007). The inflammasome: a danger sensing complex triggering innate immunity. Curr Opin Immunol.

[11] Schroder K, Zhou R, Tschopp J (2010). The NLRP3 inflammasome: a sensor for metabolic danger?. Science.

[12] Menu P, Vince JE (2011). The NLRP3 inflammasome in health and disease: the good, the bad and the ugly. Clin Exp Immunol.

[13] Iwata M, Ota KT, Duman RS (2013). The inflammasome: pathways linking psychological stress, depression, and systemic illnesses. Brain Behav Immun.

[14] Yirmiya R, Rimmerman N, Reshef R (2015). Depression as a microglial disease. Trends Neurosci.

[15] Saijo K, Glass CK (2011). Microglial cell origin and phenotypes in health and disease. Nat Rev Immunol.

[16] Tremblay ME, Stevens B, Sierra A, Wake H, Bessis A, Nimmerjahn A (2011). The role of microglia in the healthy brain. J Neurosci.

[17] Wake H, Moorhouse AJ, Miyamoto A, Nabekura J (2013). Microglia: actively surveying and shaping neuronal circuit structure and function. Trends Neurosci.

[18] Perry VH, O’Connor V (2010). The role of microglia in synaptic stripping and synaptic degeneration: a revised perspective. ASN Neuro.

[19] James ML, Belichenko NP, Nguyen TV, Andrews LE, Ding Z, Liu H, Bodapati D, Arksey N, Shen B, Cheng Z (2015). PET imaging of translocator protein (18 kDa) in a mouse model of Alzheimer’s disease using N-(2,5-dimethoxybenzyl)-2-18F-fluoro-N-(2-phenoxyphenyl)acetamide. J Nucl Med.

[20] Wang Y, Yue X, Kiesewetter DO, Niu G, Teng G, Chen X (2014). PET imaging of neuroinflammation in a rat traumatic brain injury model with radiolabeled TSPO ligand DPA-714. Eur J Nucl Med Mol Imaging.

[21] Golla SS, Boellaard R, Oikonen V, Hoffmann A, van Berckel BN, Windhorst AD, Virta J, Te Beek ET, Groeneveld GJ, Haaparanta-Solin M (2016). Parametric binding images of the TSPO ligand 18F-DPA-714. J Nucl Med.

[22] Endres CJ, Coughlin JM, Gage KL, Watkins CC, Kassiou M, Pomper MG (2012). Radiation dosimetry and biodistribution of the TSPO ligand 11C-DPA-713 in humans. J Nucl Med.

[23] Jacobson O, Kiesewetter DO, Chen X (2015). Fluorine-18 radiochemistry, labeling strategies and synthetic routes. Bioconjug Chem.

[24] Yue N, Huang H, Zhu X, Han Q, Wang Y, Li B, Liu Q, Wu G, Zhang Y, Yu J (2017). Activation of P2X7 receptor and NLRP3 inflammasome assembly in hippocampal glial cells mediates chronic stress-induced depressive-like behaviors. J Neuroinflammation.

[25] Xu Y, Bai Z, Huang Q, Pan Y, Pan D, Wang L, Yan J, Wang X, Yang R, Yang M (2017). PET of HER2 expression with a novel 18FAl labeled affibody. J Cancer.

[26] Hill MN, Hellemans KG, Verma P, Gorzalka BB, Weinberg J (2012). Neurobiology of chronic mild stress: parallels to major depression. Neurosci Biobehav Rev.

[27] Guilloux JP, Seney M, Edgar N, Sibille E (2011). Integrated behavioral z-scoring increases the sensitivity and reliability of behavioral phenotyping in mice: relevance to emotionality and sex. J Neurosci Methods.

[28] Strekalova T, Spanagel R, Bartsch D, Henn FA, Gass P (2004). Stress-induced anhedonia in mice is associated with deficits in forced swimming and exploration. Neuropsychopharmacology.

[29] Setiawan E, Wilson AA, Mizrahi R, Rusjan PM, Miler L, Rajkowska G, Suridjan I, Kennedy JL, Rekkas PV, Houle S, Meyer JH (2015). Role of translocator protein density, a marker of neuroinflammation, in the brain during major depressive episodes. JAMA Psychiatry.

[30] Barron AM, Garcia-Segura LM, Caruso D, Jayaraman A, Lee JW, Melcangi RC, Pike CJ (2013). Ligand for translocator protein reverses pathology in a mouse model of Alzheimer’s disease. J Neurosci.

[31] Daugherty DJ, Selvaraj V, Chechneva OV, Liu XB, Pleasure DE, Deng W (2013). A TSPO ligand is protective in a mouse model of multiple sclerosis. EMBO Mol Med.

[32] Wei XH, Wei X, Chen FY, Zang Y, Xin WJ, Pang RP, Chen Y, Wang J, Li YY, Shen KF (2013). The upregulation of translocator protein (18 kDa) promotes recovery from neuropathic pain in rats. J Neurosci.

[33] Girard C, Liu S, Adams D, Lacroix C, Sineus M, Boucher C, Papadopoulos V, Rupprecht R, Schumacher M, Groyer G (2012). Axonal regeneration and neuroinflammation: roles for the translocator protein 18 kDa. J Neuroendocrinol.

[34] Rupprecht R, Rammes G, Eser D, Baghai TC, Schule C, Nothdurfter C, Troxler T, Gentsch C, Kalkman HO, Chaperon F (2009). Translocator protein (18 kD) as target for anxiolytics without benzodiazepine-like side effects. Science.

[35] Martin A, Boisgard R, Theze B, Van Camp N, Kuhnast B, Damont A, Kassiou M, Dolle F, Tavitian B (2010). Evaluation of the PBR/TSPO radioligand [(18)F]DPA-714 in a rat model of focal cerebral ischemia. J Cereb Blood Flow Metab.

[36] Venneti S, Lopresti BJ, Wiley CA (2006). The peripheral benzodiazepine receptor (translocator protein 18kDa) in microglia: from pathology to imaging. Prog Neurobiol.

[37] Rupprecht R, Papadopoulos V, Rammes G, Baghai TC, Fan J, Akula N, Groyer G, Adams D, Schumacher M (2010). Translocator protein (18 kDa) (TSPO) as a therapeutic target for neurological and psychiatric disorders. Nat Rev Drug Discov.

[38] Cosenza-Nashat M, Zhao ML, Suh HS, Morgan J, Natividad R, Morgello S, Lee SC (2009). Expression of the translocator protein of 18 kDa by microglia, macrophages and astrocytes based on immunohistochemical localization in abnormal human brain. Neuropathol Appl Neurobiol.

[39] Batarseh A, Papadopoulos V (2010). Regulation of translocator protein 18 kDa (TSPO) expression in health and disease states. Mol Cell Endocrinol.

[40] Lavisse S, Guillermier M, Herard AS, Petit F, Delahaye M, Van Camp N, Ben Haim L, Lebon V, Remy P, Dolle F (2012). Reactive astrocytes overexpress TSPO and are detected by TSPO positron emission tomography imaging. J Neurosci.

[41] Colton CA (2009). Heterogeneity of microglial activation in the innate immune response in the brain. J NeuroImmune Pharmacol.

[42] Smith RS (1991). The macrophage theory of depression. Med Hypotheses.

[43] Koo JW, Duman RS (2008). IL-1beta is an essential mediator of the antineurogenic and anhedonic effects of stress. Proc Natl Acad Sci U S A.

[44] Levine J, Barak Y, Chengappa KN, Rapoport A, Rebey M, Barak V (1999). Cerebrospinal cytokine levels in patients with acute depression. Neuropsychobiology.

[45] Maes M, Bosmans E, Meltzer HY, Scharpe S, Suy E (1993). Interleukin-1 beta: a putative mediator of HPA axis hyperactivity in major depression?. Am J Psychiatry.

[46] Black R, Kronheim S, Sleath P, Greenstreet T, Virca GD, March C, Kupper T (1991). The proteolytic activation of interleukin-1 beta. Agents Actions Suppl.

[47] Kostura MJ, Tocci MJ, Limjuco G, Chin J, Cameron P, Hillman AG, Chartrain NA, Schmidt JA (1989). Identification of a monocyte specific pre-interleukin 1 beta convertase activity. Proc Natl Acad Sci U S A.

[48] Franchi L, Eigenbrod T, Munoz-Planillo R, Nunez G (2009). The inflammasome: a caspase-1-activation platform that regulates immune responses and disease pathogenesis. Nat Immunol.

[49] Vande Walle L, Lamkanfi M (2011). Inflammasomes: caspase-1-activating platforms with critical roles in host defense. Front Microbiol.

[50] Sutterwala FS, Ogura Y, Zamboni DS, Roy CR, Flavell RA (2006). NALP3: a key player in caspase-1 activation. J Endotoxin Res.

[51] Domercq M, Matute C (2004). Neuroprotection by tetracyclines. Trends Pharmacol Sci.

[52] Hinwood M, Tynan RJ, Charnley JL, Beynon SB, Day TA, Walker FR (2013). Chronic stress induced remodeling of the prefrontal cortex: structural re-organization of microglia and the inhibitory effect of minocycline. Cereb Cortex.

[53] McKim DB, Weber MD, Niraula A, Sawicki CM, Liu X, Jarrett BL, Ramirez-Chan K, Wang Y, Roeth RM, Sucaldito AD, et al. Microglial recruitment of IL-1beta-producing monocytes to brain endothelium causes stress-induced anxiety. Mol Psychiatry. 2017; 10.1038/mp.2017.64 PMC5628107 28373688

[54] Arakawa S, Shirayama Y, Fujita Y, Ishima T, Horio M, Muneoka K, Iyo M, Hashimoto K (2012). Minocycline produced antidepressant-like effects on the learned helplessness rats with alterations in levels of monoamine in the amygdala and no changes in BDNF levels in the hippocampus at baseline. Pharmacol Biochem Behav.

